# RNA Modifications in Osteoarthritis: Epitranscriptomic Insights into Pathogenesis and Therapeutic Targets

**DOI:** 10.3390/ijms26104955

**Published:** 2025-05-21

**Authors:** Shabnam Radbakhsh, Mehdi Najar, Makram Merimi, Mohamed Benderdour, Julio C. Fernandes, Johanne Martel-Pelletier, Jean-Pierre Pelletier, Hassan Fahmi

**Affiliations:** Osteoarthritis Research Unit, University of Montreal Hospital Research Center (CRCHUM), Montreal, QC H2X 0A9, Canada; shabnam.radbakhsh@umontreal.ca (S.R.); mehdi.najar@ulb.be (M.N.); makram.merimi.cri@gmail.com (M.M.); mohamed.benderdour@umontreal.ca (M.B.); julio.c.fernandes@umontreal.ca (J.C.F.); jm@martelpelletier.ca (J.M.-P.); dr@jppelletier.ca (J.-P.P.)

**Keywords:** osteoarthritis, inflammation, epitranscriptomics, RNA modification, N6-methyladenosine, 5-methylcytosine, N7-methylguanosine, 2′-O-ribose methylation

## Abstract

Osteoarthritis (OA) is a chronic joint disorder characterized by progressive degeneration of articular cartilage, pain, synovial inflammation, and bone remodeling. Post-transcriptional RNA modifications, known as epitranscriptome, are a group of biochemical alterations in the primary RNA transcript that might influence RNA structure, stability, and function. Different kinds of RNA modifications have been recognized, such as methylation, acetylation, pseudouridylation, and phosphorylation. N6-methyladenosine (m6A), 5-methylcytosine (m5C), N7-methylguanosine (m7G), 2′-O-ribose methylation (2′-O-Me), and pseudouridylation (Ψ) are the most prevalent RNA modifications. Recent studies have shown that disruption in these modifications can interfere with gene expression and protein function. Here, we will review all types of RNA modifications and how they contribute to the onset and progression of OA. To the best of our knowledge, this is the first review comprehensively addressing all epitranscriptomic modifications in OA.

## 1. Introduction

Osteoarthritis (OA) is a complex, multifactorial chronic disorder affecting the entire joint, including bone and cartilage, leading to reduced mobility and disability [1]. Based on the Global Burden of Disease 2021 estimates, OA affected 7.6% of the global population, representing approximately 595 million individuals, with a higher prevalence in women than in men [2]. Pharmacological medicines mainly provide relief from symptoms, and no definitive therapy exists to stop the progression of the disease [3]. Although the precise mechanisms underlying OA are not yet fully understood, inflammation and cartilage degeneration through matrix metalloproteinases (MMPs) enzymes and a disintegrin-like and metalloproteinase with thrombospondin type 1 motifs (ADAMTS) family proteins are the main contributors to OA pathogenesis [4,5]. Multiple molecular pathways are implicated in the development of OA. The Wnt/β-catenin and TGF-β/SMAD pathways, which regulate chondrocyte homeostasis and cartilage metabolism; the NF-κB and MAPK pathways, which mediate inflammatory responses; along with the Akt/mTOR/PI3K and oxidative stress pathways, which contribute to autophagy, chondrocyte apoptosis, and extracellular matrix (ECM) degradation, are considered among the main pathways involved in OA [6,7].

Genetics plays a significant role in the development of OA and involves the interplay of multiple genes [8,9]. Over 100 genomic risk loci associated with OA have been identified in large-scale genome-wide association studies (GWAS) [10]. Type II collagen (*COL2A1*), *COL9A2*, *COL11A1*, *COL11A2*, *COL1A1*, *COMP*, *AGC1*, and *TGFβ1*, which are related to cartilage metabolism, and inflammatory genes that encode cytokines such as interleukin-1 alpha (*IL1A*) and *IL1B*, are key genes related to OA [11,12,13]. Epigenetic factors, which involve heritable alterations in DNA structure without changing the primary DNA sequence, also interact with genetic risk loci and contribute to the pathogenesis of OA [14]. Recent studies on the epigenetics of OA have focused on DNA methylation, histone modifications, and non-coding RNAs. DNA methylation affects the expression of key genes such as *SOX9*, *IL1B* [15], *15-LOX*, and *DP1* [16,17]. Histone modifications (H3K9me3, H3K27me3, and H3K4me3) also influence chromatin organization and the expression of OA-related genes [18,19,20]. Non-coding RNAs, including microRNAs (e.g., miR-7, miR-136), long non-coding RNAs (lncRNAs) such as HOTAIR and MALAT1, and circular RNAs (circRNAs) like circ_0136474, are additional components of the epigenetic regulatory mechanisms in OA [21]. In addition to epigenetic changes at the DNA level, modifications at the RNA level may also play a role in the pathogenesis of OA.

Epitranscriptomic modifications refer to reversible biochemical alterations, such as methylation, acetylation, pseudouridylation, and phosphorylation, that occur in RNA after transcription [22]. These modifications influence RNA structure, stability, and localization, which are involved in the regulation of gene expression [23]. Several studies indicate that disruptions in RNA modification processes may contribute to the pathogenesis of different diseases; however, despite growing interest, their role in OA pathogenesis remains unclear [24]. In this article, we aim to review epitranscriptomic studies related to OA to elucidate the roles of these modifications in the onset and/or progression of the disease. To date, most review articles in this field have focused on epigenetic modifications [18,25,26,27] or exclusively on m6A [28]. To the best of our knowledge, this is the first comprehensive review to address all types of epitranscriptomic modifications investigated in the context of OA.

## 2. Epitranscriptomic Modifications

**Figure 1:**
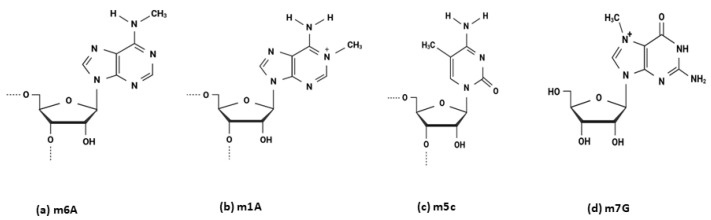
Representative chemical structures of common RNA methylation modifications: (**a**) N6-methyladenosine (m6A), (**b**) N1-methyladenosine (m1A), (**c**) 5-methylcytidine (m5C), and (**d**) 7-methylguanosine (m7G), highlighting the positions where methyl groups are added to the respective nucleosides.

Epitranscriptomic modifications refer to the post- or co-transcriptional alterations in all types of RNA, including coding messenger RNA (mRNA) and non-coding RNA, such as structural and regulatory RNAs [29]. To date, over 170 distinct types of chemical modifications have been identified in all types of living organisms [30]. Various biochemical groups, such as methyl and acetyl groups, can be enzymatically added to different nucleotides within the RNA structure. For instance, N6-methyladenosine (m6A) is a modification in which a methyl group is added to the nitrogen at the sixth position of the adenine base in RNA. Similarly, N1-methyladenosine (m1A) refers to methylation at the nitrogen at position 1 of adenine, 5-methylcytidine (m5C) involves methylation at the carbon at position 5 of cytidine, and N7-methylguanosine (m7G) is characterized by methylation at the nitrogen at position 7 of guanosine (Figure 1) [31].

**Figure 2:**
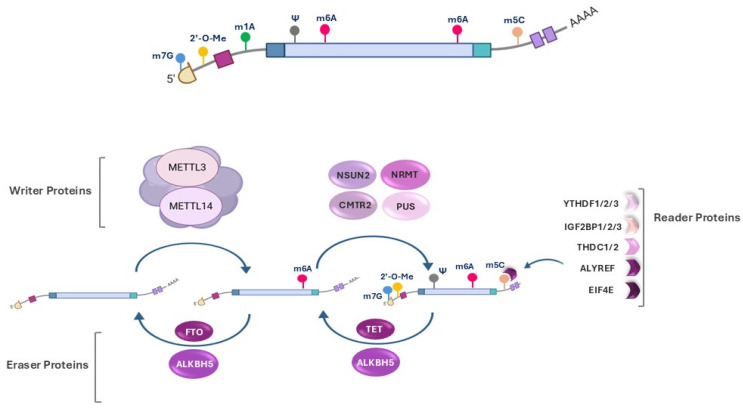
Schematic representation of RNA modifications and their regulatory proteins. The diagram illustrates the dynamic regulation of RNA modifications by writer, eraser, and reader proteins. Writer proteins (e.g., METTL3, METTL14, NSUN2, NRMT, CMTR1, and PUS) catalyze the addition of modifications such as m6A, m5C, ψ, and m7G. Eraser proteins (e.g., FTO, ALKBH5, and TET) remove specific modifications, and reader proteins (e.g., YTHDF1/2/3, IGF2BP1/2/3, THOC1/2, ALYREF, and EIF4E) recognize these modifications and regulate RNA function and cellular responses.

Recent advances in high-throughput methods have identified substantial amounts of epitranscriptome sequencing data, enabling the detection of both known and novel RNA modification sites [32]; however, the exact prevalence, biological roles, and functional mechanisms of many RNA modifications remain unclear [33]. Radioactive labeling and bisulfite sequencing were the earliest techniques utilized to identify adenine-specific tRNA methylase activity and detect cytosine methylation (5-methylcytosine) positions. Subsequently, chromatographic separation and mass spectrometry allowed for the isolation and precise quantification of methylated nucleotides in mRNA and small RNAs, such as miRNAs. In addition, the combination of immunoprecipitation with next-generation sequencing has facilitated high-resolution mapping of modifications. Furthermore, cryo-electron microscopy (Cryo-EM) and protein nanopore-based sequencing, along with bioinformatics tools like MeTDiff, have significantly improved the differential analysis of RNA modifications in recent years [34]. RNA modifications are catalyzed by RNA-modifying proteins (RMPs), which can be classified into three main groups: “the writers”, “the readers/ modifiers”, and “the erasers” [35,36,37] (Figure 2).

**Writers.** “Writers” are enzymes responsible for catalyzing RNA modification reactions by adding chemical groups such as methyl, acetyl, or pseudouridine to specific nucleotides or functional sites within the RNA molecule [38]. These enzymes demonstrate specificity based on the type of modification and the target RNA molecule. Several key writer enzymes are critical for RNA modifications, regulating RNA metabolism, stability, and decoding efficiency. Methyltransferase-like 3 (METTL3) and METTL14 constitute the core of the m6A methyltransferase complex, catalyzing m6A, the most prevalent internal mRNA modification. Wilms tumor 1-associated protein (WTAP) functions as a regulatory subunit, facilitating the m6A reaction by interacting with METTL3/METTL14. NSUN2 and DNMT2 are key m5C writer enzymes, contributing to RNA stability and function. The RNMT/RAM complex, along with the METTL1/WDR4 complex, serves as a writer enzyme for the m7G modification, which plays a crucial role in RNA capping and translation regulation. Additionally, PUS1, PUS7, and PUS10 mediate pseudouridylation (Ψ), a modification that enhances RNA structural integrity and functionality [35].

**Readers.** The second group of RNA modifiers consists of “readers” that specifically recognize and bind to chemically modified nucleotides in RNA molecules to interpret the regulatory roles of RNA modifications by mediating various biological processes, such as translation initiation, mRNA decay, and splicing [38]. For instance, YTH domain-containing proteins and the IGF2BP1/2/3 family act as readers in m6A modifications and regulate mRNA stability, decay, and translation efficiency. ALYREF and YBX1 are key readers in m5C modifications, which promote nuclear export and mRNA stabilization. EIF4E is a key reader of m7G modifications, which enhance cap-dependent translation initiation. PUS-binding proteins are recognized as readers of Ψ modifications, which can specifically recognize pseudouridylated sites and mediate the interaction with regulatory factors that modulate RNA processing, localization, stability, or translation [39].

**Erasers.** The final group of enzymes, known as “erasers”, is responsible for removing chemical modifications that influence RNA metabolism [38]. In m6A modification, fat mass and obesity-associated protein (FTO) and AlkB Homolog 5 (ALKBH5) serve as key demethylases, and regulate RNA stability, splicing, and translation. In m5C modification, the ten-eleven translocation (TET) enzyme family, particularly TET2, is implicated in the oxidative demethylation of m5C residues, thereby affecting RNA processing [40]. To date, no specific eraser enzyme has been identified for the m7G modification. Similarly, no dedicated eraser has been characterized for 2′-O-methylation, and due to its stable chemical structure, it is likely to represent an irreversible modification [41,42].

These chemical modifications and their enzymes are essential regulators of the gene expression process. They are involved in RNA processing steps, including RNA splicing, transcript stability, translation, and RNA–protein interactions [43]. Therefore, dysregulation in these processes has been implicated in the development of various human diseases [44].

## 3. Epitranscriptomic Modifications in OA

Among the various types of RNA modifications identified, m6A, m5C, m7G, Ψ, and 2′O have been extensively studied [45]. Although it remains unclear whether epitranscriptomic modifications per se contribute to disease pathogenesis [24], several studies have identified dysregulated expression patterns of their modifiers in OA tissues [46,47]. In a bioinformatic study conducted on OA datasets collected from the Gene Expression Omnibus (GEO), 12 genes involved in epitranscriptomic RNA modifications were identified. Among these, *METTL3* was upregulated, while *ALKBH1*, *EIF3*, *IGF2BP3*, and *YTHDC1* were downregulated [46]. However, another bioinformatic investigation reported that METTL3 levels were lower in OA patients than in healthy controls [48]. Interestingly, a genetic epidemiology study suggests that *METTL3* gene polymorphisms contribute to the susceptibility to knee OA (KOA) in individuals from Southern China. The rs1061026 variant was linked to a higher risk, whereas rs1139130 and rs1263802 were associated with a decreased risk of KOA [49]. Additional studies showed that the level of YTHDF2, a crucial m6A regulator, was decreased [50], while YTHDF3 and IGFBP2 were upregulated in OA patients compared to healthy groups [51]. In conclusion, the expression levels of writer enzymes such as METTL3 are elevated in OA patients compared to healthy individuals, and increased RNA modifications, particularly methylation, appear to be involved in OA pathogenesis; therefore, inhibiting these alterations may represent a potential therapeutic approach. In the following sections, we will discuss various types of RNA modifications identified in OA, beginning with m6A, which is the most well-characterized modification affecting both coding and non-coding RNAs.

## 4. N6-Methyladenosine (m6A)

N6-methyladenosine (m6A) refers to an epigenetic change characterized by the transfer of a methyl group at the N-6 position of adenosine in RNA molecules. This modification typically occurs in the 3′-untranslated regions (UTRs) near stop codons and follows the consensus sequence RRACH (R: G or A; H: A, C, or U) [52,53]. This highly conserved process is conducted by a multicomponent enzyme complex (MTC), which includes methyltransferases (m6A writers) responsible for adding the methyl group, demethylases (m6A erasers) that remove it, and regulatory proteins (m6A readers) that recognize and bind to the m6A modification site in RNA [54]. METTL3, as the key catalytic component, METTL14 as a stabilizing supplement for METTL3, and Wilms tumor suppressor-1-associated protein (WTAP), as a regulatory element, are considered the main methyltransferases [52]. Additional writers have been identified in recent years, including METTL5 [55], METTL16 [56], KIAA1429 [57], and RBM15 [58]. YTHDF1, YTHDF2, YTHDF3, YTHDC1, and YTHDC2, containing the YTH domain (YT521-B homology), are the most important readers involved in m6A methylation, with YTHDF1 and YTHDF3 promoting translation, YTHDF2 mediating mRNA degradation, YTHDC1 facilitating nuclear export, and YTHDC2 functioning as a helicase [59,60]. Finally, FTO and ALKBH5 are two main demethylases belonging to the dioxygenase family of enzymes, which remove m6A methylation [54]. In this part, we will discuss the effects of m6A modification on mRNA transcripts, microRNAs, long non-coding RNAs, and circular RNAs in OA pathogenesis [50].

### 4.1. Impact of m6A Modification on OA-Related mRNAs

m6A modification regulates the expression of genes involved in various processes recognized for their role in the pathogenesis of OA, such as inflammation, ECM degeneration, ferroptosis, pyroptosis, cellular senescence, and autophagy (Table 1) [31,61,62].

#### 4.1.1. Inflammation

Inflammation plays a crucial role in the progression of OA and is influenced by both joint-resident and systemically derived immune cells [63]. Damage-associated molecular patterns (DAMPs) activate innate immune receptors such as toll-like receptors (TLRs) and NOD-like receptors (NLRs), leading to local inflammation and unexpected flare-ups. Several studies have reported that altered expression of m6A RNA modification regulators, including METTL3, YTHDC1, and FTO, affects inflammatory events and the regulation of the immune microenvironment. This is especially true for macrophage polarization, the transition from the M1 to M2 phenotype, and the infiltration and activation of various immune cells, including T cells, B cells, and dendritic cells [50,51,64,65,66]. Bioinformatics analysis identified 122 differentially expressed m6A-related genes in OA. Among them, YTHDF2, a key m6A reader involved in RNA degradation, was significantly downregulated. Its reduced expression promotes pro-inflammatory M1 polarization and inhibits anti-inflammatory M2 polarization [64]. An in vivo study in 6–8-week-old C57BL/6 mice confirmed that YTHDF2 promotes *p53* mRNA degradation in M2 macrophages and suppresses NF-κB, p38, and JNK signaling pathways in M1 macrophages, thereby modulating inflammatory gene expression [64]. Analysis of gene expression data from 63 OA and 59 healthy samples in the GEO database, along with validation in 10 OA and 10 normal tissue samples, revealed altered expression of YTHDF2 and IGFBP2 in OA compared to healthy samples. YTHDF2 was downregulated, leading to a decrease in regulatory T cells (Tregs). Conversely, IGFBP2 was upregulated, which negatively affected dendritic cells (DCs) and decreased DCs in OA tissues [51]. IGF2BP3 is another regulator of RNA modification implicated in OA pathogenesis. Analysis of patient-derived samples showed upregulation of six m6A regulators in OA synovium, including FTO, YTHDC1, METTL5, IGF2BP3, ZC3H13, and HNRNPC. Further mechanistic investigations demonstrated that IGF2BP3 upregulation promotes synovial inflammation by enhancing M1 macrophage polarization, in contrast to the activity of YTHDF2, and has an indirect relationship with M2 polarization [67]. Variations in *METTL3* genes, another key regulator, have been associated with an increased risk of OA, mainly by influencing the shift from the pro-inflammatory M1 phenotype to the anti-inflammatory M2 phenotype. Bioinformatic analysis also confirmed that *METTL3* was downregulated in OA patients, while *FTO* was upregulated, both contributing to inflammation and immune cell infiltration in OA patients [48]. WTAP, a co-writer in m6A modification, activates the Wnt/β-catenin pathway by stabilizing its inhibitor, *FRZB* mRNA, through an m6A-dependent mechanism. Notably, the Wnt/β-catenin pathway contributes to the production of proinflammatory cytokines such as IL-6 and TNF-α. Thus, it may enhance inflammation and sustain inflammatory responses [68,69]. ALKBH8 and YTHDF3 were also significantly upregulated in OA patients. Conversely, WDR4, an m7G modifier, as well as RPUSD4, PUS1, NUDT21, and FBL, were downregulated compared to healthy individuals. These molecular changes are closely related to immuno-inflammatory regulation, particularly involving the infiltration of activated B cells, activated CD8+ T cells, type II helper T cells, natural killer (NK) cells, and eosinophils [70,71,72]. Therefore, it is possible that m6A modification contributes to enhanced inflammation in OA by regulating immune cell polarization, promoting pro-inflammatory responses, and disrupting immune homeostasis.

#### 4.1.2. ECM Degradation

ECM degradation is the main hallmark of OA, which is mediated by important enzymes such as MMPs and ADAMTSs [73,74]. Different MMPs contribute to cartilage ECM degradation and tissue remodeling, such as collagenases (e.g., MMP-1, MMP-13), gelatinases (e.g., MMP-2, MMP-9), and stromelysins (e.g., MMP-3, MMP-10) [75]. RNA modification, particularly m6A methylation, modulates the expression of genes involved in cartilage ECM degradation [61]. In line with this, reduced *METTL3* expression and m6A methylation have been observed in IL-1β-treated SW1353 cells and OA cartilage samples. Overexpression of *METTL3* via a lentiviral vector regulates MMP-1, MMP-3, MMP-13, and TIMP-1/2 at both mRNA and protein levels. Therefore, METTL3 may be involved in the pathogenesis of OA by regulating the balance between matrix metalloproteinases and their inhibitors, and thus affecting cartilage ECM homeostasis [61]. In another study, silencing of METTL3 by using specific shRNA in an OA mouse model decreased the levels of MMP-13 and increased collagen type II (COL2A1) levels [76]. To better understand the role of METTL3, mechanical tension was applied to chondrocytes using the FX-5000 Tension System. The results demonstrated that METTL3 mediated *SOX9* mRNA methylation and reduced its stability, which in turn suppressed *COL2A1* expression and decreased ECM synthesis [77]. Furthermore, targeting METTL3 in a mouse model of OA and chondrocyte culture increased the levels of COL2A1 and aggrecan, while ADAMTS5 and MMP-13 levels decreased, significantly alleviating OA symptoms [78]. METTL3 also enhances the methylation of *STAT1* mRNA, resulting in increased stability and translation. STAT1 regulates the expression of immune and inflammatory genes, including ADAMTS12, which encodes a metalloprotease involved in ECM degradation. Elevated STAT1 activity promotes ADAMTS12 expression, contributing to ECM degeneration and inflammation in OA [79]. Therefore, the METTL3/STAT1 axis might be an important target for novel therapeutic strategies in OA treatment.

Ribosomal protein L38 (Rpl38) and suppressor of cytokine signaling 2 (Socs2) are two other proteins that might be implicated in the degradation of ECM and can be modified by METTL3 activity. In OA cartilage, *RPL38* is upregulated while *SOCS2* is downregulated. Silencing *RPL38*, by using si-RPL38, increases SOCS2 expression through METTL3-mediated methylation, modulating the JAK/STAT3 pathway and reducing apoptosis and ECM degeneration [80]. These findings indicate that targeting *RPL38/SOCS2* may offer a promising therapeutic strategy for OA management.

METTL3 not only regulates mRNAs under OA conditions but also impacts ECM degradation-related gene expression in Kashin–Beck disease (KBD), a type of osteoarthropathy with characteristics similar to OA. Combined m6A MeRIP-Seq and RNA-Seq analysis of peripheral blood samples from three KBD patients identified six hypermethylated and upregulated genes, and 23 hypomethylated and downregulated genes compared to controls [81]. Trichothecene mycotoxin-2 (T-2 toxin) has an important role in KBD progression. It has also been shown that T-2 toxin regulates m6A modification of *Ctsk* mRNA, a cysteine protease involved in bone and joint disorders, by contributing to ECM degeneration. Therefore, METTL3 may influence the disease process by interacting with T2 toxin and the methylation of the *Ctsk* mRNA gene [82]. Besides METTL3, WTAP may also play a role in ECM degradation, leading to OA [83]. Additional studies have demonstrated that carbonic anhydrase XII (CA XII/12) enzyme levels are increased in the knee cartilage of OA patients [83,84]. WTAP enhances *CA12* mRNA stability via m6A methylation, promoting oxidative stress, inflammation, apoptosis, and cartilage degeneration in the destabilization medial meniscus (DMM) mouse models. Silencing *CA12* using short hairpin RNA (shRNA) reduces ROS and MDA production, along with increasing catalase (CAT) and superoxide dismutase (SOD) activities, which ultimately attenuate apoptosis and ECM degradation [83,85]. As previously mentioned, the Wnt/β-catenin pathway is implicated in OA through the regulation of various mechanisms. FRZB, a key inhibitor of this pathway, is reduced in OA. WTAP destabilizes *FRZB* mRNA in an m6A-dependent manner, leading to Wnt/β-catenin activation, cartilage degradation, and OA progression [68]. Besides writers, demethylase enzymes, known as erasers, and proteins that function as readers also contribute to disease conditions. In two separate studies, conducted using a murine model of intervertebral disc disease (IVDD) [86] and an MIA (monosodium iodoacetate) mouse model simulating OA [87], it was found that ALKBH5 induces demethylation in the 3′-UTR region of *Runx2* mRNA, enhancing its stability in a YTHDF1-dependent manner. The increased level of Runx2 leads to the upregulation of ADAMTSs and MMPs, ECM degradation, and the progression of IVDD and OA. Therefore, the demethylation of *Runx2* mRNA might be associated with ECM degradation and contribute to OA pathogenesis [86,87]. During OA, m6A modification is upregulated due to the increased activity of methylase enzymes, while the expression of the demethylase FTO is decreased. Under normal conditions, RUNX1 regulates FTO expression by binding to specific sites on the FTO promoter, stabilizing SAMD2, and regulating TGF-β signaling. However, in OA tissues, the dysfunction of RUNX1-mediated FTO transcription leads to decreased FTO expression, which in turn causes reduced demethylation and instability of *SMAD2* mRNA, leading to TGF-β signaling disruption [88]. Interestingly, chondrocytes from heterozygous FTO-cKO mice showed increased levels of MMP-3 and MMP-13, while COL2A1, Aggrecan, and SOX9 protein expression were significantly decreased. Therefore, FTO influences OA by modulating ECM degradation and synthesis through its effects on SAMD2 and other key genes [88].

#### 4.1.3. Ferroptosis

Ferroptosis, a type of regulated cell death caused by iron-dependent lipid peroxidation, is one of the fundamental mechanisms involved in OA [89]. RNA modifications and their related regulators modulate this process [62,90,91]. During ferroptosis, glutathione (GSH) levels decline due to glutathione peroxidase 4 (GPX4) inhibition, leading to iron-dependent ROS accumulation, increased chondrocyte susceptibility to oxidative stress, and ECM degradation. In IL-1β-stimulated chondrocytes, elevated METTL14 and MMP-13 levels are associated with reduced GPX4 and collagen II expression [62,91]. Knockdown of METTL14 using three siRNAs in primary mouse chondrocytes inhibited ferroptosis and ECM degradation by suppressing m6A methylation of *GPX4* mRNA [62]. Similar to METTL14, METTL3 influences ferroptosis in chondrocytes during KOA. Ferroptosis activators such as Erastin, RSL3, sorafenib, and FIN56 elevate the level of high mobility group box 1 (HMGB1) via METTL3-mediated m6A methylation, contributing to cartilage damage and pain. Both in vitro and in vivo studies demonstrated that knocking down *METTL3* in IL-1β-treated cells can significantly reduce HMGB1 levels, thereby inhibiting ferroptosis and attenuating inflammation, cartilage damage, and pain [90]. YTHDF1, a reader, also modulates ferroptosis in bone and joint diseases. It has been shown that YTHDF1 regulates ferroptosis in NPCs by upregulating SLC7A11 and through its involvement in GSH synthesis. Overexpression of YTHDF1, along with Hypoxia-Inducible Factor-1 (HIF-1), suppresses NPC ferroptosis by enhancing SLC7A11 protein expression after binding to *SLC7A11* mRNA [91].

#### 4.1.4. Pyroptosis

Pyroptosis is another type of programmed cell death triggered by inflammation, and evidence of this process, such as increased levels of pyroptosis-related cytokines, has been detected in OA chondrocytes [92]. Six RNA modification-related genes (*ADAMDEC1*, *IGHM*, *OGN*, *TNFRSF11B*, *SCARA3*, and *PTN*), along with six hub genes linked to pyroptosis and RNA modification, including *CXCL10*, *CXCL9*, *CCR7*, *CCL5*, *CXCL1*, and CCR2, were recognized as potential biomarkers for the pathogenesis of OA and RA [31]. Epitranscriptomic RNA modifications, such as methylation, influence pyroptosis in OA and other bone diseases [93,94]. NIMA-related kinase 7 (NEK7), a key regulator of NLRP3 inflammasome activation, is increased in OA samples. Inhibition of METTL3 expression modulates m6A modification and reduces *NEK7* mRNA levels, thereby suppressing chondrocyte pyroptosis and slowing OA progression in both in vitro and in vivo models [93].

#### 4.1.5. Cellular Senescence

Cellular senescence plays a significant role in the aging process and contributes to the development of OA [95,96]. A comprehensive study utilizing three models of cellular senescence demonstrated that the m6A methylation process, assisted by the IGF2BP1 reader, enhances mesenchymal stem cells (MSCs) senescence by affecting the stability of *CYP1B1* mRNA, which is a member of the cytochrome P450 superfamily with monooxygenase activity. Moreover, CYP1B1 was identified as an important downstream target of ALKBH5, such that its overexpression improved MSC efficacy and decreased cell senescence in an OA mouse model [95]. In another osteoporosis animal model, IGF2BP2 expression increased in aging osteoblasts under mechanical stress and H_2_O_2_ exposure, which promotes osteoblast senescence by improving the stability of *Slc1a5* mRNA and inhibiting cell cycle progression. Furthermore, it has been found that overexpression of METTL3 promotes osteoblast senescence, while treatment with Cpd-564, a specific METTL3 inhibitor, along with IGF2BP2 siRNA, leads to increased bone mass and decreased senescence in aged rats [97].

#### 4.1.6. Autophagy

Autophagy is an important process in the pathogenesis of OA [96]. Although autophagy acts as a protective mechanism in the early stages by preventing cell death, uncontrolled autophagic activity in advanced OA may lead to cellular damage [98]. M6A has been shown to modulate the expression of autophagy-related (*ATG*) genes by affecting mRNA splicing, stability, and degradation [99]. Aged fibroblast-like synovial cells (FLSs) induce chondrocyte senescence, contributing to cartilage damage. Impaired autophagy in OA-FLS increases senescence-associated secretory phenotype (SASP), worsening joint degeneration. METTL3-mediated m6A modification decreases the stability of autophagy-related 7 (*ATG7*) mRNA, which promotes FLS senescence. Silencing *METTL3* improves autophagic flux, decreases SASP, and relieves cartilage destruction in a mouse OA model [100]. Moreover, Lv et al. [101] reported that chondrocyte-derived exosomes contribute to OA via the long non-coding RNA OANCT, which binds to FTO and activates the PI3K/AKT/mTOR pathway. This inhibits autophagy and promotes M1 macrophage polarization, leading to increased inflammation and cartilage degradation [101]. These findings highlight the complex interplay between m6A modification and autophagy in OA, suggesting that precise regulation of this pathway could hold therapeutic potential for mitigating OA.

**Table 1:** Effects of m6A Modification on mRNAs Involved in OA Pathogenic Pathways.

Pathogenic Mechanism	Target of Modification	Models	Effect on OA Pathogenesis	References
Inflammation	mRNA (*IL-6*, *IL-8*, *IL-12*, and *TNF-α*)	In vitro: IL-1β-treated mouse chondrocyte cell line ATDC5 In vivo: Collagenase-induced OA mouse model	METTL3 regulates OA progression by modulating pro-inflammatory factors, such as IL-6, IL-8, IL-12, and TNF-α, and NF-κB signaling	[76]
	mRNA (*NLRP3*)	In vivo: DMM-induced OA mouse model In vitro: IL-1β-treated human OA primary chondrocytes	Targeting METTL3 via miR-1208 decreases *NLRP3* mRNA levels and activity, thereby reducing inflammatory factor levels	[78]
ECM Degradation	mRNA (*MMP-1*, *MMP-3*, *MMP-13*, *TIMP-1*, *TIMP-2*)	In vitro: IL-1β-treated human chondrocyte Human OA cartilage explants	METTL3 overexpression modulates ECM degradation by modulating the TIMP/MMP balance	[61]
	mRNA (*MMP-13*, and *COL2A1*)	In vitro: IL-1β-treated mouse chondrocyte cell line ATDC5 In vivo: Collagenase-induced OA mouse model	METTL3 deletion reduces *MMP-13* expression and enhances that of *COL2A1*	[76]
	mRNA (*SOX9*, *COL2A1*)	Human OA cartilage explants	METTL3 regulates *SOX9* expression and suppresses *COL2A1* levels	[77]
	mRNA (*COL2A1*, *ADAMTS5* and *MMP-13*)	In vivo: DMM-induced OA mouse model Human OA cartilage explants	METTL3 inhibition increases COL2A1 and aggrecan levels while decreasing ADAMTS5 and MMP-13 expression	[78]
	mRNA (*STAT1*)	In vitro: IL-1β-treated rat primary chondrocytes In vivo: ALCT induced OA rat model Human OA cartilage explants	METTL3 enhances the expression of *STAT1*, and upregulates *ADAMTS12*	[79]
	mRNA (*RPL38* and *SOCS2*)	In vitro: IL-1β-treated human OA primary chondrocytes In vivo: DMM-induced OA mouse model Human OA cartilage explants	Silencing *RPL38* upregulates *SOCS2* expression via METTL3-mediated methylation	[80]
	mRNA ((*Ctsk*)	In vitro: IL-1β-treated rat primary chondrocytes In vivo: T-2 toxin-induced cartilage injury rat model	T-2 toxin induces cartilage damage via METTL3-m6A methylation of Ctsk	[82]
	mRNA (*CA12*)	In vitro: IL-1β-treated human chondrocyte Human normal and OA cartilage explants	WTAP enhances *CA12* mRNA stability and leads to chondrocyte apoptosis	[83]
	mRNA (*FRZB*)	In vivo: DMM-induced OA mouse model Human OA cartilage explants	WTAP-mediated m6A modification reduces *FRZB* expression, and activates the Wnt/β-catenin pathway	[68]
	mRNA (*Runx2*)	In vitro: LPS-treated mouse primary NP cells In vivo: LPS-induced IVDD mouse model, MIA-induced OA rat model	ALKBH5 facilitates *Runx2* mRNA demethylation and upregulates MMPs and ADAMTSs	[86,87]
	mRNA (*SMAD2*)	In vitro: IL-1β-treated mouse primary chondrocytes In vivo: DMM-induced OA mouse model Human OA cartilage explants	Decreased levels of FTO stabilizes *SMAD2* mRNA and regulates TGF-β signaling	[88]
Ferroptosis	mRNA (*GPX4*)	In vitro: IL-1β-treated mouse primary chondrocytes In vivo: MIA-induced OA rat model	Silencing METTL14 suppresses *GPX4* mRNA m6A modification and inhibits ferroptosis	[62]
	mRNA (*HMGB1*)	In vitro: IL-1β-treated rat primary chondrocytes In vivo: MIA-induced OA rat model	METTL3 promotes m6A methylation of *HMGB1* mRNA, and increases ferroptosis	[90]
	mRNA (*SLC7A11*)	In vivo: Acupuncture-induced IVDD rat model	YTHDF1 and HIF-1α overexpression reduces NPC ferroptosis by promoting SLC7A11 translation	[91]
Pyroptosis	mRNA (*NeK7*)	In vitro LPS-treated human OA primary chondrocytes In vivo: DMM-induced OA mouse model Human OA cartilage explants	METTL3 knockdown reduces *NEK7* mRNA levels, and suppresses chondrocyte pyroptosis	[93]
Cell senescence	mRNA (*SLC1A5*)	In vitro: H_2_O_2_-treated primary rat osteoblasts In vivo: si-Igf2bp2 and Cpd-564-treated osteoporosis rat model	METTL3 and IGF2BP2 inhibition modulates the METTL3/IGF2BP2-SLC1A5 axis and reduces osteoblast senescence	[97]
	mRNA (*CYP1B1*)	In vitro: Human normal cartilage explants In vivo: ALCT induced OA mice model	m6A methylation stabilizes *CYP1B1* mRNA and promotes MSC senescence	[95]
Autophagy	mRNA (*ATG7*)	In vivo: DMM-induced OA mouse model Human normal cartilage explants	METTL3-mediated m6A modification reduces *ATG7* mRNA and impairs autophagy	[100]
	mRNA (*PIK3R5*)	In vitro: IL-1β-treated rat primary chondrocytes In vivo: MIA-induced OA rat model Human OA cartilage explants	FTO activates the PI3K/AKT/mTOR axis	[101]

This table summarizes the role of m6A modification in OA, emphasizing its impact on key pathogenic mechanisms such as inflammation, extracellular matrix degradation, ferroptosis, pyroptosis, cell senescence, and autophagy. It includes the modified targets (proteins, mRNA, and signaling pathways), study model (in vitro or in vivo), and their effects on the pathogenesis of OA.

### 4.2. Impact of m6A Modification on Non-Coding RNAs

In addition to mRNAs, m6A modification also targets non-coding RNAs, including miRNAs, lncRNAs, and circRNAs involved in OA (Table 2).

#### 4.2.1. MicroRNAs (miRNAs)

MiR-126-5p plays a crucial role in suppressing the PI3K/Akt signaling pathway in OA pathogenesis [102]. METTL3, in association with the protein DiGeorge syndrome critical region gene 8 (DGCR8), modulates the maturation of miR-126-5p and suppresses the PI3K/Akt signaling pathway. However, when METTL3 is inhibited, the maturation of miR-126-5p is disrupted, which allows the PI3K/Akt pathway to function normally. This contributes to the suppression of inflammation and ECM repair [102]. WTAP may also be involved in OA by regulating miRNAs, such as miR-92b-5p [103]. A study using human OA chondrocytes found that WTAP promotes the maturation of pri-miR-92b into miR-92b-5p, thereby enhancing its inhibitory effects on *TIMP4* mRNA. This process facilitates the degradation of *TIMP4* mRNA. Together, these data indicate that WTAP and its target microRNA, pri-miR-92p, are involved in the pathogenesis of OA [103]. In addition to methylases, m6A demethylase enzymes such as FTO also modulate miRNAs and contribute to OA pathogenesis. It has been demonstrated that FTO levels are decreased in LPS-treated C28/I2 cells and rat models of OA. In interaction with the protein DGCR8, this methylase modulates pri-miR-515-5p activity in an m6A-dependent manner. Since TLR4 is a direct target of miR-515-5p, the MyD88/NF-κB pathway is activated, leading to inflammation and apoptosis. Conversely, overexpression of FTO represses the TLR4/MyD88/NF-κB axis, thereby attenuating OA pathogenesis [104]. FTO also regulates the maturation of pri-miR-3591 through demethylation and decreases its inhibitory effect on *PRKAA2* mRNA. Increased PRKAA2 levels stimulate the AMP-activated protein kinase (PRKAA1/2), also known as the AMPK pathway, which mitigates cartilage damage by promoting cell proliferation and suppressing apoptosis [105].

#### 4.2.2. Long Non-Coding RNAs (lncRNAs)

LncRNAs are another group of target molecules for RNA modifications, which can serve as underlying mechanisms in OA pathogenesis. Mechanistic analysis in IL-1β-treated chondrocytes revealed that the m6A methyltransferase METTL3 methylates LINC00680, which leads to its upregulation. LINC00680 interacts with *SIRT1* mRNA by binding to the m6A site on its 3′-UTR, improving its stability. It is noteworthy that SIRT1 plays a protective role in OA by exhibiting anti-catabolic, anti-inflammatory, anti-oxidative stress, and anti-apoptotic effects. Therefore, METTL3 may regulate OA progression by modulating the LINC00680/SIRT1 axis [106]. In contrast to LINC00680, ENST00000512512.1, also known as IGFBP7-OT, is another lncRNA upregulated in OA and positively correlated with the severity of cartilage damage. M6A modification enhances IGFBP7-OT levels by regulating the DNMT1/DNMT3a-IGFBP7 axis and DNA methylation processes. The increase in IGFBP7 significantly suppresses chondrocyte viability, stimulates chondrocyte apoptosis, and leads to the degradation of ECM components. Therefore, targeting IGFBP7-OT could be a promising therapeutic strategy for treating OA [107]. Research conducted using a mouse model of OA showed that METTL14 also plays a role in modulating the expression of LncRNAs involved in ECM degeneration. METTL14 facilitated cartilage damage in OA mice by upregulating FAS-AS1 and activating JAK/STAT3 signaling. FAS-AS1 increased *ADAM8* mRNA stability by binding to FMR1, which in turn activates JAK/STAT3 signaling, leading to OA chondrocyte apoptosis and ECM degradation [108].

LncRNA NORAD is upregulated in NPCs in the IVDD cell model. WTAP, in association with YTHDF2, enhances the m6A modification of NORAD, which interacts with PUM1/2, RNA-binding proteins that operate as negative regulators of gene expression. PUM1/2 represses the expression of target E2F transcription factor 3 (*E2F3*) mRNA, thereby promoting cellular senescence and contributing to IVDD progression [109].

Furthermore, m6A demethylases such as FTO, like methylases, regulate OA by modulating lncRNAs. AC008440.5 (AC008) is highly expressed in OA cartilage and contributes to chondrocyte apoptosis, ECM degradation, and OA progression by suppressing the miR-328-3p/ANKH pathway. Importantly, FTO downregulates AC008 transcription through demethylation, leading to increased miR-328-3p levels, specifically targeting AQP1 and ANKH. This regulatory mechanism enhances chondrocyte viability and reduces apoptosis and ECM degradation [110]. ALKBH5 is another m6A demethylase that regulates lncRNA expression and activity. HS3ST3B1-IT1 is decreased in OA, and its overexpression has been shown to ameliorate MIA-induced OA in rats [111]. This lncRNA belongs to the 3-O-sulfotransferase (3-OST) family, a group of enzymes involved in heparan sulfate (HS) biosynthesis and interactions with key proteins that regulate cartilage homeostasis. ALKBH5 enhances HS3ST3B1-IT1 RNA stability and expression through demethylation in human OA cartilage [111]. Moreover, it has been demonstrated that ALKBH5-mediated demethylation of lncRNAs positively modulates the osteogenic differentiation of human adipose-derived stem cells (hASCs). ALKBH5 regulates the AK311120 lncRNA, which interacts with RNA-binding proteins such as DExH-box helicase 9 (DHX9) and YTHDC2. This interaction mediates the downregulation of mitogen-activated protein kinase 7 (MAP2K7) and the JNK signaling pathway, promoting osteogenesis [112]. The m6A demethylation of lncRNAs may play an important role in the pathogenesis of IVDD. ZFP217, in association with the FTO, demethylates lncRNA LOC102555094, which in turn activates the miR-431/GSK-3β/Wnt pathway. This activation disrupts glucose metabolism in NP cells, contributing to the development of IDD [113].

#### 4.2.3. Circular RNAs (circRNAs)

Recent studies have shown that circular RNAs (circRNAs) are implicated in the pathogenesis of OA [114]. circMYO1C is an m6A-modified circRNA with m6A characteristics that increase in OA in vitro/in vivo models, and its knockdown suppresses inflammatory factors such as TNF-α, IL-6, and IL-8. This circRNA targets the circMYO1C/m6A/HMGB1 axis and upregulates its stability and levels, contributing to inflammation and chondrocytes apoptosis in OA [115]. In contrast to circMYO1C, circRERE levels are decreased in OA. This reduction is likely due to increased m6A modifications on circRERE, which makes it more prone to degradation by the YTHDF2-HRSP12-RNase P/MRP complex. The breakdown of circRERE dysregulates its downstream miR-195-5p/IRF2BPL/β-catenin pathway, leading to an imbalance in β-catenin that may contribute to OA development [116]. Concerning the impact of m6A on circRNAs associated with proliferation, in vitro observations in antler chondrocyte cell culture demonstrated that the m6A reader YTHDC1 facilitates the nuclear export of circRNA3634 in an m6A-dependent manner. This circRNA, by binding to miR-124486-5, regulates MAPK1 expression and enhances the proliferation and migration of antler chondrocytes [114].

**Table 2:** Effects of m6A Modification on Non-Coding RNAs Involved in OA.

Type of Non-Coding RNAs	Target of Modification	Models	Effect on OA Pathogenesis	References
Micro RNAs (miRNAs)	miR-126-5p	In vitro: IL-1β-treated human OA primary chondrocytes Human OA cartilage explants	METTL3 regulates miR-126-5p maturation and inhibits the PI3K/Akt axis	[102]
	miR-92b-5p	Human OA cartilage explants	WTAP enhances miR-92b-5p maturation, thereby reducing TIMP4 expression	[103]
	pri-miR-515-5p	In vitro: LPS-treated human chondrocyte In vivo: MIA-induced OA rat model	FTO modulates pri-miR-515-5p activity and regulates the TLR4/MyD88/NF-κB pathway	[104]
	pri-miR-3591	Human OA cartilage explants	FTO regulates pri-miR-3591 maturation, and suppresses its inhibitory effects on PRKAA2	[105]
Long non-coding RNAs (lncRNAs)	lncRNA: LINC00680	In vitro: IL-1β-treated human chondrocytes	METTL3 upregulates LINC00680, which interacts with *SIRT1* mRNA	[106]
	lncRNA: IGFBP7-OT	In vitro: LPS-treated human chondrocytes In vivo: Mouse model of MIA-induced OA Human OA cartilage explants	METTL3-mediated m6A modification of IGFBP7-OT regulates the DNMT1/DNMT3a-IGFBP7 axis	[107]
	lncRNA: FAS-AS1	In vitro: IL-1β-treated human chondrocytes In vivo: ALCT induced OA rat model	METTL14 upregulates FAS-AS1, and activates the JAK/STAT3 signaling pathway	[108]
	lncRNA: NORAD	Human normal and DDD NP tissues	WTAP-mediated m6A modification of NORAD regulates the PUMILIO/E2F3 pathway, promoting senescence	[109]
	lncRNA: AC008	In vitro: IL-1β-treated human normal primary chondrocytes In vivo: MIA-induced OA rat model Human normal and OA cartilage explants	FTO suppresses AC008 transcription, thereby elevating miR-328-3p levels and downregulating AQP1 and ANKH	[110]
	lncRNA: HS3ST3B1-IT1	In vivo: MIA-induced OA mouse model Human normal and OA cartilage explants	ALKBH5-mediated demethylation stabilizes HS3ST3B1-IT1 RNA	[111]
	lncRNA: AK311120	In vitro: Human adipose-derived stem cells (hASCs) In vivo: Mouse model of mandibular defect	ALKBH5 demethylates lnc-AK311120, regulates MAP2K7/JNK signaling, and promotes osteogenesis	[112]
	lncRNA: LOC102555094	In vivo: Acupuncture-induced IVDD rat model	Demethylation of LOC102555094 by ZFP217 and FTO activates the miR-431/GSK-3β/Wnt pathway, which promotes IVDD	[113]
Circular RNAs (circRNAs)	circRNA: circMYO1C	Human normal and OA cartilage explants	An m6A-modified circMYO1C enhances *HMGB1* mRNA stability, promoting chondrocyte apoptosis	[115]
	circular RNA: circRERE	In vitro: IL-1β-treated human chondrocytes In vivo: DMM-induced OA mouse model	m6A modification promotes circRERE degradation and contributes to OA pathogenesis	[116]

This table summarizes the effects of m6A modification on non-coding RNAs in OA. It includes the modified targets (microRNAs, long non-coding RNAs, circular RNAs), study models (in vitro, in vivo), and their effects on OA.

## 5. 5-Methylcytosine (m5C)

5-Methylcytosine (m5C) is another post-transcriptional RNA methylation that occurs in various types of RNA, including mRNA, tRNA, and rRNA, though it is most prevalent in tRNAs [117]. Furthermore, m5C modifications are found in long non-coding RNAs, circular RNAs, and small nuclear RNAs [40]. The modified site in mRNA is mainly located in the 3′ untranslated region (3′UTR), in tRNA at the junction between the variable loop and the T stem-loop, and in rRNA within the 18S and 25S rRNAs [118,119]. The m5C writers, which add a methyl group (-CH_3_) to the 5th carbon of cytosine, include the NOL1/NOP2/SUN domain (NSUN) family and DNA methyltransferase 2 (DNMT2). The ten-eleven translocation (TET) family proteins are proposed as m5C erasers, which oxidize m5C into cytosine-5-hydroxymethylation (hm5C) in RNA, particularly mRNA. YBX1 and ALYREF are recognized as m5C readers that can identify methylated cytosines in GC-rich regions of RNA [120]. The m5C modification is involved in RNA processing steps, including tRNA stability and rRNA assembly [121]. Alterations in m5C methylation have been shown to affect various biological processes such as cell proliferation, apoptosis, and aging [121]. Using MeRIP-Seq, Yu and colleagues demonstrated that m5C modification affects the expression of 133 genes related to OA. Among these, MMP-14 and COL1A1 are identified as hub genes involved in collagen and ECM degradation, suggesting their potential role in OA pathogenesis and as therapeutic targets [122]. In another study, data from an OA cohort obtained from the Gene Expression Omnibus, m5C regulators were found to be differentially expressed in OA compared to normal samples, which might serve as novel biomarkers for OA diagnosis. Moreover, Gene Ontology (GO) and the Kyoto Encyclopedia of Genes and Genomes (KEGG) enrichment analyses indicated that some of these genes were involved in endoplasmic reticulum (ER) stress, mitochondrial autophagy, and immune infiltration [123]. Notably, CRISPR screening on mitochondrial RNA in HEK-293T cells has indicated that NSUN4 might be a key regulator of mt-dsRNA expression [124]. Regarding the impact of m5C on cell differentiation, it has been demonstrated that NSUN4, along with METTL3, regulates the chondrogenic differentiation of BMSCs through methylation of the 3′ UTR of *Sox9* mRNA, which promotes cartilage repair and provides a potential therapeutic target for clinical applications [125].

## 6. N7-Methylguanosine (m7G)

M7G is a highly conserved RNA modification involving the addition of a methyl group to the N7 position of guanosine either at the 5′ cap of mRNA, as the m7G cap, or at internal positions within mRNAs, tRNAs [126]. The RNMT/RAM complex is the primary m7G methyltransferase responsible for adding the m7G cap to mRNAs in humans, while the METTL1/WDR4 complex acts as a writer enzyme for m7G modification at internal RNA sites, particularly in tRNAs [127]. Nevertheless, m7G erasers and readers have not yet been fully identified. Similar to other types of modifications, m7G plays a crucial role in RNA metabolic processes, including splicing, stability, transcription, translation, and nuclear export [41]. Recent studies have demonstrated that m7G modification may be implicated in different diseases, including OA [128]. Analysis of three GEO datasets identified eighteen m7G-related regulatory factors in OA, among which nine M7G regulators exhibited statistically significant differences. Hao et al. identified four key m7G regulators, including EIF4E2, DCP2, SNUPN, and LARP1, as potential biomarkers for OA. They designed a diagnostic model based on these four genes, which successfully distinguished OA patients from healthy individuals and differentiated early- from end-stage OA. Additionally, they developed an m7G scoring system that correlated with variations in the immune microenvironment, suggesting a functional role for m7G methylation in OA pathogenesis [128]. In two other in silico studies, NUDT11, NUDT1, SNUPN, METTL1, EIF4E2, and CYFIP1 m7G-related hub genes were identified as biomarkers for the early diagnosis and prognosis of OA, which may be associated with immune cell infiltration and inflamed phenotypes [129,130]. In vitro and in vivo studies revealed that METTL1 and m7G levels are markedly elevated in OA chondrocytes. This increase upregulates mitochondrial transfer RNA (mt-tRF3b-LeuTAA) expression, which in turn decreases SENP1 levels and increases SIRT3 SUMOylation, eventually inhibiting PINK1/Parkin-mediated mitochondrial mitophagy. The disruption in mitochondrial function contributes to cartilage degradation, and using a PMC-tRF3b-LeuTAA inhibitor in vivo attenuates cartilage damage in a mouse model [127]. These findings suggest that targeting the METTL1/m7G/mt-tRF3b-LeuTAA axis could be a promising strategy for OA treatment.

## 7. Pseudouridylation (Ψ)

Pseudouridylation is a post-transcriptional RNA modification in which uridine (U) is converted to pseudouridine (Ψ) through the activity of pseudouridine synthases (PUSs). These enzymes can function independently or in combination with small nucleolar RNAs (snoRNAs) that guide site-specific modifications. Similar to other modifications, pseudouridylation occurs in all types of RNAs, including mRNAs, rRNAs, tRNAs, and snRNAs [131]. Pseudouridylation plays various physiological roles depending on the RNA it modifies, but it is most commonly associated with the regulation of gene expression. The incorporation of Ψ into RNA increases the rigidity of the phosphodiester backbone, which in turn affects the molecule’s thermodynamic stability and spatial conformation, leading to enhanced stability, particularly in shorter RNA species [132]. A recent study indicates that OA alters specific pseudouridylation sites in ribosomal RNA. Specifically, the reduction of SNORA33 decreased modification at the 28S-ψ4966 site, which influences ribosome function and the cellular proteome [133].

## 8. 2′-O-Ribose Methylation (2′-O-Me)

2’-O-ribose methylation is a chemical modification that involves adding a methyl group to the 2’-hydroxyl group of the ribose sugar in RNA. This modification is commonly found in rRNAs and tRNAs and plays a key role in stabilizing RNA structure, promoting proper folding, and enhancing RNA function [134]. It has been found that rRNA modifications, especially 2’-O-methylation (2’-O-Me), are altered in chondrocytes exposed to OA synovial fluids. Specifically, reduced 2’-O-Me at the U14 site in 5.8S rRNA, which was mediated by SNORD71 knockdown, led to changes in ribosome function and increased type I collagen production [135]. These findings suggest that altered 2’-O-Me plays a role in OA progression by affecting the translation of key ECM proteins.

## 9. Polyadenylation

Polyadenylation involves the addition of adenosine nucleotides, known as the poly(A) tail, to the 3′ end of most eukaryotic mRNA molecules [136]. Although this co-/post-transcriptional process is not typically considered an epitranscriptomic modification since it regulates the stability and functionality of mRNA. Two separate studies reported that polyadenylation site alterations are not prevalent in OA cartilage [137,138]. Yet, treatment with cordycepin (3′ deoxyadenosine), a polyadenylation inhibitor, was found to reduce Runx2, MMP-3/-13, and ADAMTS-4 levels, leading to attenuated inflammation, ECM degeneration, and pain in rodent models of OA [139]. The role of alternative polyadenylation in OA was investigated using QuantSeq Rev. While differential gene expression confirmed OA-related biological processes, only 20 transcripts showed significant changes in polyadenylation, primarily affecting the length of the 3′ UTR and the stability of mRNA, particularly in transcripts of the oncostatin M receptor (OSMR) gene, which is involved in cartilage degradation and inflammation [138]. Table 3 provides an overview of m5C, m7G, 2′-O-methylation, and Ψ RNA modifications.

## 10. Therapeutic Potential of Targeting RNA Modifications in OA

OA currently lacks an effective cure, and most available medications primarily aim to relieve pain and manage symptoms. Emerging strategies such as epigenetic modulation, tissue-engineered cartilage implants, MSC therapy, and targeted intra-articular biologic drugs offer an innovative and promising approach for the treatment of OA [140,141,142,143,144,145,146,147]. RNA-targeted therapies are a rapidly growing and promising area of research and therapeutic development.

As mentioned before, M6A is the most prevalent internal modification in eukaryotic mRNA, and increasing evidence indicates that it plays important roles in various cellular processes involved in the pathogenesis of OA, including ECM degradation, regulation of inflammatory responses, senescence, ferroptosis, and autophagy. On the other hand, numerous studies reported aberrant expression of enzymes that catalyze, recognize, or remove m6A [28,48,51]. Therefore, m6A modifications and m6A-related enzymes may constitute novel therapeutic targets in the treatment of OA. For instance, silencing of METTL3 and inhibition of m6A were shown to be protective in collagenase-induced OA in mice [76]. In another study, overexpression of FTO attenuated OA in a rat model of MIA-induced OA [104]. Importantly, some natural products used in the management of OA were reported to induce their beneficial effects via modulation of m6A methylation. For example, Bushen Huoxue Decoction (BSHXD), a traditional Chinese medicine used to relieve OA symptoms, prevents LPS-induced NLRP3 inflammasome activation and pyroptosis by reducing m6A modification in cultured chondrocytes [148]. Triptolide (TP), another bioactive compound extracted from the herb *Tripterygium wilfordii* Hook F, with anti-inflammatory and anti-arthritic properties. Treatment with TP reduced IGF2BP3 expression in vitro and showed a strong binding affinity in molecular docking, suggesting that TP may exert its effects by targeting m6A-related post-transcriptional regulation [149]. Finally, Gubi decoction (GBD), a traditional herbal medicine known for its virtues in the management of OA, was protective in DMM-induced OA in mice via promoting chondrocyte autophagy and suppressing METTL3-dependent ATG7 m6A methylation [150].

Together, these data suggest that m6A modification can serve as a potential target in OA. Further studies are needed to fully understand the therapeutic potential of targeting m6A modification in OA.

## 11. Conclusions

In conclusion, emerging evidence indicates that epitranscriptomic modifications profoundly contribute to the pathogenesis of OA by modulating key cellular and molecular processes. These modifications, including m6A, m5C, m7G, Ψ, and 2′-O methylation, have been shown to affect all types of RNA, such as mRNA and non-coding RNA, by influencing their structure, stability, localization, and function. The altered expression of RNA-modifying enzymes, such as METTL3, YTHDF2, and ALKBH1, influences chondrocyte homeostasis, inflammatory signaling, ECM integrity, programmed cell death (ferroptosis and pyroptosis), cellular senescence, and autophagy. For instance, dysregulated m6A methylation mediated by the METTL3/METTL14 complex and removed by FTO and ALKBH5, reduces the stability and translation of key transcripts such as SOX9, STAT1, RUNX2, and GPX4, thereby promoting cartilage degradation and synovial inflammation. Similarly, altered m5C and m7G modifications affect collagen remodeling and mitochondrial function, while alterations in pseudouridylation and 2′-O methylation impact ribosomal accuracy and ECM protein synthesis. Targeting the epitranscriptomic machinery offers potential for attenuating or reversing disease progression. Small-molecule inhibitors and PROTACs targeting methyltransferases (e.g., METTL3, METTL14) and demethylases (e.g., FTO, ALKBH5), along with oligonucleotide-based methods to modulate RNA–reader interactions, have demonstrated efficacy in preclinical models of OA. Future research will need to focus on uncovering the mechanistic details of RNA modifications in different cell types and contexts, particularly between healthy and osteoarthritic joints. Integrating multi-omics and single-cell epitranscriptomic profiling will identify new targets and optimize therapeutic approaches.

## Figures and Tables

**Table 3:** Effects of m5C, m7G, 2′-O-ribose methylation, and pseudouridylation modifications in OA.

Type of Modifications	Target of Modification	Models	Effect on OA Pathogenesis	References
m5C Methylation	mRNA (*Sox9*)	In vivo: Surgically induced full-thickness osteochondral defect model in rat	NSUN4 methylates the 3′ UTR of *Sox9* mRNA, regulating chondrogenic differentiation	[125]
m7G Methylation	Mitochondrial transfer RNA (mt-tRF3b-LeuTAA)	In vitro: IL-1β-treated human OA primary chondrocytes In vivo: DMM-induced OA mouse model	METTL1-mediated m7G modification promotes mt-tRF3b-LeuTAA, SIRT3 SUMOylation, and cartilage degeneration	[127]
Pseudouridylation	rRNA (at sites 28S- 4966)	In vitro: IL-1β-treated human normal primary chondrocytes	OA microenvironment alters site-specific changes in rRNA pseudouridylation	[133]
2′-*O*-ribose methylation (2′-*O*-Me)	rRNA (U14in 5.8S)	In vitro: IL-1β-stimulated human chondrocytes	Reduced 2′-O-Me of U14 in 5.8S rRNA impairs ribosome function and contributes to OA pathogenesis	[135]
Polyadenylation	mRNA (*OSMR*)	Human OA cartilage explants	Disruption of polyadenylation alters 3′UTR length, stabilizes *OSMR* mRNA, and promotes ECM degradation	[138]
	mRNA (*Runx2*, *MMPS-3/-13*, *ADAMTS-4*)	In vivo: DMM-induced OA mouse model Human OA cartilage explants	Cordycepin inhibits polyadenylation, and reduces Runx2 and MMP-3/-13 levels	[139]

This table summarizes the m5C, m7G, 2′-O-ribose methylation and pseudouridylation effects in OA. It includes the modified targets (different types of RNAs), study models (in vitro or in vivo), and their effects on disease progression.

## Data Availability

No new data were generated or analyzed in this study. All data discussed are cited from previously published sources and are included in the manuscript.

## Abbreviations

2′-O-Me	2′-O-ribose methylation
ADAMTS	A Disintegrin and Metalloproteinase with Thrombospondin Motifs
ALKBH5	AlkB Homolog 5
ECM	Extracellular Matrix
EGFR	Epidermal Growth Factor Receptor
EIF4E	Eukaryotic Translation Initiation Factor 4E
EIF4E2	Eukaryotic Translation Initiation Factor 4E2
hm5C	Cytosine-5-Hydroxymethylation
IGF2BP1	Insulin-like Growth Factor 2 Binding Protein 1
IL-12	Interleukin 12
IL1 α	Interleukin 1 Alpha
IL1β	Interleukin 1 Beta
IL-6	Interleukin 6
IL-8	Interleukin 8
IVDD	Intervertebral Disc Degeneration
KOA	Knee Osteoarthritis
lncRNA	Long Non-Coding RNA
m1A	Methylation of Adenosine at Position N1
m5C	5-methylcytosine
m6A	N6-methyladenosine
m7G	N7-methylguanosine
MeRIP-Seq	Methylated RNA Immunoprecipitation Sequencing
METTL	Methyltransferase-Like
miCLIP	Methylation-induced Crosslinking and Immunoprecipitation
miRNA	MicroRNA
mRNA	Messenger RNA
ROS	Reactive Oxygen Species
rRNA	Ribosomal RNA
SONFH	Steroid-Associated Osteonecrosis of the Femoral Head
SUMOylation	Small Ubiquitin-like Modifier Modification
TET	Ten-Eleven Translocation
TGFβ1	Transforming Growth Factor Beta 1
tRNA	Transfer RNA
VDR	Vitamin D Receptor
Ψ	Pseudouridylation
OA	Osteoarthritis
PI3K/AKT/mTOR	Phosphoinositide 3-Kinase/Protein Kinase B/Mechanistic Target of Rapamycin
WTAP	Wilms Tumor 1-Associated Protein
YTH	YTH Domain-Containing Proteins
ZFP217	Zinc Finger Protein 217
